# Editorial: Early diagnosis of kidney disease in young adulthood

**DOI:** 10.3389/fneph.2025.1700226

**Published:** 2025-10-06

**Authors:** Antonino Sidoti, Vincenzo Panichi

**Affiliations:** ^1^ Nephrology Department, Unità Sanitaria Locale Sud Est Toscana, Arezzo, Italy; ^2^ Department of Clinical and Experimental Medicine, University of Pisa, Pisa, Italy

**Keywords:** CKD screening, CKD - chronic kidney disease, genetics, hypertension, IgA nephropathy, Alport, hemodialysis

We have found that 25% of people arriving for dialysis have no specific diagnosis of the type of kidney disease they are affected by. A debate has arisen: is case-finding in high-risk subjects, such as those with a family history of kidney disease or other risk factors, or population-wide screening more advisable? The former approach targets individuals who are more likely to have the disease, while the latter aims to identify cases in the entire population. Population-wide screening seems advisable in low-income countries where transplant and hemodialysis are not widely available due to a lack of economic resources and expertise. In high-income countries, population-wide screening would be cost-effective if there is a high prevalence of chronic kidney disease (CKD) (1). The last years have shown a progressively diminishing age cost-effectiveness in participants in population-wide screenings. There is a two-way connection between CKD and cardiovascular risk (1).

Efforts are being made in dialysis to prevent high mortality, which is currently 22.83% at 4 years, mainly due to cardiovascular and nutritional risk, in patients aged 18-59; there is a suggestion to analyze CKD approaches at a country-wide level (Sun et al.).

Data on Alport disease (2, 3) and IgA nephropathy (2, 4) indicate that these diseases can be detected, even if it is not currently possible to do so at the individual level, in the general population through the coupling of biomarkers and genetic information work-up. Mutations in *COL4A3*, *COL4A4*, or *COL4A5* have a range of phenotypic effects that span from Alport syndrome to thin basement membrane nephropathy, passing through focal glomerulosclerosis or no disease at all. Early diagnosis of these mutations and clinical follow-up could help elucidate the mechanisms of disease. An early diagnosis allows for cost-effective, kidney-preserving treatment, such as SGLT2 inhibitors (5), ARBs, ACE inhibitors, and, in the future, non-steroidal Aldosterone receptor blockers. In IgA, early detection could aid in the use of new therapies (6); targeting specific kidney diseases could help treat larger populations promptly.

Proteinuria is a crucial component of CKD screening. In a quest for accuracy, 24-hour urine collection and the urinary creatinine/albumin ratio have made way for formulas adapted to the population; these have been developed to replace 24-hour urine collection with a method that shows better performance in people younger than 65 years with a urinary protein/creatinine ratio less than 500 mg/g, making it amenable to use in young population for the sake of screening (Jia et al.). There is an incentive to develop formulas specific to a determined population, refraining from using 24-hour collection in extensive population studies.

Measurement of blood pressure is a mainstay of CKD screening. In children and adolescents, elevated blood pressure levels increase the long-term risk of major adverse kidney events (7).

A comprehensive phenotypic description of the disease (Song et al.) enables us to go beyond the already known effects of the mutation; in this case, a complex kidney and reproductive dysfunction is discovered at age 23, starting from a diagnosis of maturity onset diabetes of the young (MODY), emphasizing the urgency and significance of early and timely diagnosis of CKD. 

Early screening for CKD not only enables a more precise classification of the disease beyond the sole kidney biopsy but also paves the way for personalized care and immunosuppressive therapy. This approach enables a follow-up procedure that empowers the nephrologist to make a significant impact on the patient’s care. The goal is to prevent the patient with the kidney disease from requiring dialysis and transplant, slowing down the progression of kidney failure, treating concomitant complications, and, when substitutive therapy is mandatory, allowing the patient to arrive for treatment in the best condition possible.

Knowledge of disease mechanisms at the cellular level is of foremost importance to slow the progression of kidney failure, i.e., stabilizing the glomerular filtration rate (GFR) over time: Chiang et al. have studied low brain-derived neurotrophic factor, a crucial player in energy homeostasis, already known to be associated with non-diabetes mellitus CKD, demonstrating its relevance also in diabetes mellitus-related CKD with low levels connected with high level of vascular adhesion molecule-1 (VCAM-1)—a factor involved in in attachment of white blood cells to the inner lining of blood vessels.

Artificial intelligence could help us identify, with the aim of CKD screening, which data are more predictive of the future development of CKD. We could achieve this goal in a big database, finding the best blend of parameters coming from anamnestic data, blood routine, and urinalysis (8).

## References

[1] Vassalotti JA Francis A Soares Dos Santos AC Jr. Correa-Rotter R Abdellatif D Hsiao LL. Are your kidneys ok? Detect early to protect kidney health. Kidney Int. (2025) 107:370–7. doi: 10.1016/j.kint.2024.12.006, PMID: 39984248

[2] Hoefele J Rao J Mallett AJ. Editorial: Genetics and epigenetics of chronic kidney disease. Front Med. (2023) 10:451. doi: 10.3389/fmed.2023.1078300, PMID: 36860336 PMC9969120

[3] Kashtan CE. Alport syndrome: Achieving early diagnosis and treatment. Am J Kidney Dis. (2021) 77:272–9. doi: 10.1053/j.ajkd.2020.03.026, PMID: 32712016

[4] Kamal F Kim J Lafayette R. Current biomarkers of igA nephropathy. Semin Nephrol. (2024) 44:540–50. doi: 10.1016/j.semnephrol.2025.151572, PMID: 40087126

[5] Cusick MM. Population-wide screening for chronic kidney disease: A cost-effectiveness analysis. Ann Internal Med. (2023) 176:788–97. doi: 10.7326/M22-3228, PMID: 37216661 PMC11091494

[6] Lemley KK Glassock RJ. Springtime for new IgA Nephropathy therapy. New Engl J Med. (2024) 390:80–1. doi: 10.1056/NEJMe2312300, PMID: 38169494

[7] Hussain J Georgieva K Robinson CH Jeyakumar N Smith G Brady T. Long-term kidney outcomes in children and adolescents with hypertension: a propensity-matched cohort study. Lancet Child Adolesc Health. (2025) 9:553–64. doi: 10.1016/S2352-4642(25)00127-0, PMID: 40578375

[8] He J Wang X Zhu P Wang X Zhang Y Zhao J. Identification and validation of an explainable early-stage chronic kidney disease prediction model: a multicenter retrospective study. Lancet. (2025) 2025:553–64. doi: 10.1016/j.eclinm.2025.103286, PMID: 40567347 PMC12192353

